# Influence of surface texturing and coatings on mechanical properties and integration with bone tissue: an in silico study

**DOI:** 10.3389/fbioe.2024.1439262

**Published:** 2024-09-02

**Authors:** Abdulkhaliq Ali F. Alshadidi, Vamsi Krishna Dommeti, Lujain Ibrahim N. Aldosari, Saeed Awod Bin Hassan, Abdulmajeed Okshah, Ali Merdji, Sandipan Roy

**Affiliations:** ^1^ Allied Dental Health Sciences Department, College of Medical Applied Sciences, King Khalid University, Abha, Saudi Arabia; ^2^ Department of Mechanical Engineering, SRM Institute of Science and Technology, Chennai, India; ^3^ Prosthodontics Department, College of dentistry, King Khalid University, Abha, Saudi Arabia; ^4^ Department of Restorative Dental Sciences “RDS” College of Dentistry, King Khalid University, Abha, Saudi Arabia; ^5^ Department of Mechanical Engineering, University of Mascara, Mascara, Algeria

**Keywords:** textured implant, hybrid coatings, bone quality, microstrain, FEA

## Abstract

**Introduction:**

This investigation delves into the mechanical behaviour of titanium dental implants, a preferred choice for tooth replacement due to their superior reliability over alternative materials. The phenomenon of implant loosening, frequently induced by masticatory activities, underscores the significance of surface modification or texturing to bolster the interaction between the implant and bone tissue. This research comprehensively examines the effects of four distinct surface texturing techniques and five varied bone quality conditions on the biomechanical performance of these implants.

**Methods:**

The scope of this study is delineated by its focus on implants of diameters 4 mm and 6 mm, with lengths measuring 9 mm and 12 mm respectively. Furthermore, the analysis incorporates the evaluation of four different coatings—hydroxyapatite, HA3TO, HA3Sr, and HA1.5TO1.5Sr—to investigate their efficacy in enhancing the osseointegration process on textured surfaces of dental implants.

**Results:**

The experimental design entails the assessment of stress distribution within the implant and its coatings, alongside the strain exerted on the surrounding cancellous bone, under the conditions of an average vertical biting force. A comparative analysis between solid implants and those subjected to surface texturing techniques has been conducted. This comparison elucidates the advantageous microstrain profiles presented by certain textured surfaces, which are deemed more conducive to optimal osseointegration.

**Discussion:**

Notably, across all examined textures, the application of hydroxyapatite (HA) and a modified HA composition (HA1.5TO1.5Sr) demonstrates significant improvements in mechanical stability, particularly in scenarios involving weak and very weak bone conditions. This study's findings contribute to the ongoing advancement in dental implant technology, emphasizing the critical role of surface texturing and coating strategies in promoting implant longevity and integration within the biomechanical environment of the human oral cavity.

## 1 Introduction

Dental implants are strategically inserted into the toothless areas of a patient’s mouth to replace missing teeth (Jemat et al., 2015). By employing three-dimensional (3D) modeling and finite element (FE) analysis, it is possible to accurately forecast the stress distribution within both the implants and the interface between the implants and the bone (Gupta et al., 2021). Implant dimensions are a significant factor in relation to bone quality, quantity, and bone mineral density (Haba et al., 2012; Toniollo et al., 2012; Dommeti et al., 2023a). Ensuring primary interfacial stability is crucial for dental implants to achieve a greater level of osseointegration (Meyer et al., 2004). Given the significance of osseointegration and primary stability, it is crucial to modify the implants through surface texturing (Bächle and Kohal, 2004). This technique enhances cell proliferation on the implant surface, leading to improved interaction. The design of implants with changed surfaces is crucial for achieving optimal osseointegration (Albrektsson et al., 1981). The increase in surface roughness and mechanical characteristics has enhanced the connection between bone and implant surfaces by promoting bone ingrowth (Jemat et al., 2015). A clinical study demonstrated that the mechanical adhesion between bone and the implant surface can be enhanced by improving the surface topology of the implant (Djebbar et al., 2010; Gupta et al., 2021). Finite element analysis (FEA) has been employed to assess the correlation between interfacial shear strength and various texturing shapes (Çelen et al., 2011). In recent studies, researchers have been investigating several surface textures, including honeycomb, spherical, diagonal, and moon patterns, using finite element (FE) modeling. Among these, the honeycomb structure shows superior performance than the others (Çelen and Özden, 2012). Additionally, sandblasting and acid etching techniques were utilized to modify the surface of the titanium implant in order to create the required surface topology (Kim et al., 2006). Texturing treatments were also applied at the micro and nano levels. Studies have demonstrated that laser-based texturing with various surface textures yields superior shear strength results compared to plain and sand-blasted surfaces on titanium material (Koshy and Tovey, 2011; Maressa et al., 2015). Using micro-groove tools resulted in the creation of significant surface roughness, leading to enhanced surface quality of titanium. This improvement in surface quality contributed to a higher level of infection prevention (Xie et al., 2012; Chouirfa et al., 2019). Hydroxyapatite (HA) is a commonly utilized ceramic substance for coating bioceramics. HA [Ca5(PO4)3OH] is a type of calcium phosphate molecule that exhibits superior stability in bodily fluids than other calcium phosphate compounds (Pramanik et al., 2007; Arnould et al., 2009). The inclusion of a modest amount of tantalum in the HA composite film has been found to exhibit improved biocompatibility and corrosion resistance (Sato et al., 2005). The primary function of the tantalum and its oxide covering is to enhance the corrosion resistance, bioactivity, and biocompatibility of orthopedic implants, hence protecting them (Xu et al., 2018). Tantalum oxide coating has been utilized on several titanium and alloy-based implants to enhance their corrosion, wear resistance, and adhesive qualities with the bone and the implant (Li et al., 2013). Within the specified timeframe of 6 weeks, dental implants coated with strontium ions exhibited a notable increase in bone growth, as indicated by previous research (Zhang et al., 2016). Several investigations have shown that strontium does not exhibit any hazardous effects (Andersen et al., 2013). A robust enhancement in the success rate of dental implants was found by applying a strontium (Sr) coating that promoted the development of new bone tissue. Based on these studies, we have chosen to focus on using strontium and tantalum minerals to promote bone formation in our current research.

The mechanical properties of dental implants are influenced by both the quality of the surrounding bone and the roughness of the implant surface. There is a need to bridge the gap in understanding the impact of texturing with coating materials on bone quality (Taharou et al., 2020; Ouldyerou et al., 2022). Hence, the primary aim of this work is to examine the mechanical impact of various surface texturing methods on the implant surface using different types of hybrid coating materials. Additionally, it seeks to assess the impact of hybrid coating on bone quality when using implants. Multiple studies have examined the effects of applying a single material coating on the smooth surfaces of Sr, silver (Ag), zinc (Zn), and magnesium (Mg) implants. However, it is crucial to comprehend the influence of hybrid coating on the surface of the textured implant. Hence, it is necessary to determine the impact of hybrid coating on both the textured implant surface and bone quality.

The aim of this study is to investigate the impact of hybrid coatings and surface texturing on the biomechanical properties of dental implants with different sizes and lengths. This study suggests that surface alterations, specifically the addition of texture to the implant surface, are crucial in improving cell adherence and the overall mechanical stability of the implant in the mouth. The dominant theory in the field suggests that the main purpose of implant surface texturing is to enhance the bond between the implant and bone tissue, thus enhancing osseointegration.

An essential element in this process is the increase in surface roughness caused by the use of certain coatings, which is believed to greatly improve osteoblast activity. The underlying assumption is that a surface with a rough texture offers a greater surface area for osteoblasts to attach to, which could result in a stronger and quicker fusion of the implant with the bone. Previous studies in this field have found a connection between the biomechanical characteristics of dental implants and two important factors: the quality of the adjacent bone and the level of surface roughness of the implant.

This study aims to enhance the current understanding of the impact of hybrid coatings and strategic texturing of implant surfaces on the biomechanical integration and stability of dental implants through a thorough investigation. The objective of this research is to both confirm earlier discoveries and enhance them by providing a detailed comprehension of how various elements interact to influence the outcome of dental implantation treatments.

In the above context, the current objectives of the study are as follows:

1. To study the mechanical response of various surface texturing methods on titanium dental implants.
2. To study the influence of different hybrid coating materials, including hydroxyapatite, HA3TO, HA3Sr, and HA1.5TO1.5Sr, on the performance of dental implants.
3. To investigate the biomechanical performance of textured dental implants under five distinct bone quality conditions.
4. To compare the stress distribution within the implant and the strain on the surrounding cancellous bone under an average biting force.
5. To identify the textured surfaces and hybrid coatings that provide optimal microstrain on a textured dental implant with hybrid coating for a better osseointegration process.

Research has shown that using micro and nano-scale surface texturing can greatly improve the mechanical interaction between the implant and the bone tissue. Research has also demonstrated that surface texturing enhances the attachment, growth, and specialization of cells, all of which are crucial for successfully integrating bone with an implant.

## 2 Materials and methods

### 2.1 Modeling implants with varying diameters and lengths

**FIGURE 1:**
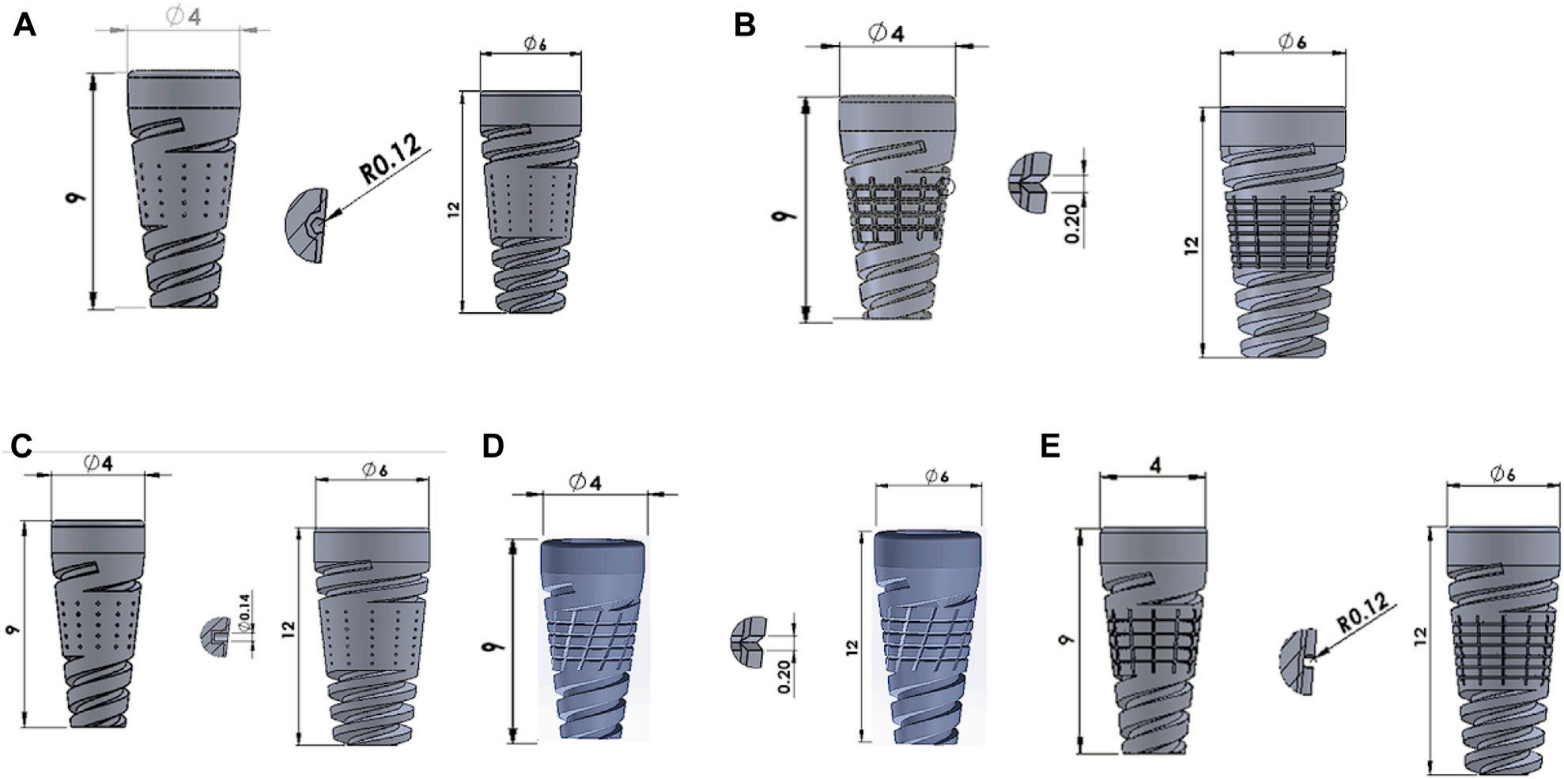
Surface modified implants with **(A)** Dome-shape texture, **(B)** V-shape texture, **(C)** Straight-shape texture, **(D)** X-shape texture, and **(E)** U-shape texture.

SOLIDWORKS 2017 (Dassault Systemes, United States) modeled the mandibular bone, which is 24.2 mm in height and 16.3 mm in width. We have modeled both the cancellous and cortical regions (Kayabaşı et al., 2006). We have used a commercial implant with a minimum diameter of 4 mm and a maximum diameter of 6 mm; we considered minimum lengths of 9 mm and 12 mm. The five distinct surface textures include dome, straight, V-, X-, and U-shape surface textures (Dommeti et al., 2021), as shown in Figure 1.

In this study, we have investigated the effect of coating materials like hydroxyapatite alone, a hybrid coating with 3% tantalum and 97% hydroxyapatite (HA3TO), a coating with 3% strontium with 97% hydroxyapatite (HA3SR), and a coating with 1.5% tantalum, 1.5% strontium, and 97% hydroxyapatite (HA1.5TO1.5Sr). The reason for selecting tantalum and its derivatives is that they possess remarkable antibacterial and osteogenic characteristics (Wang et al., 2021).

**FIGURE 2:**
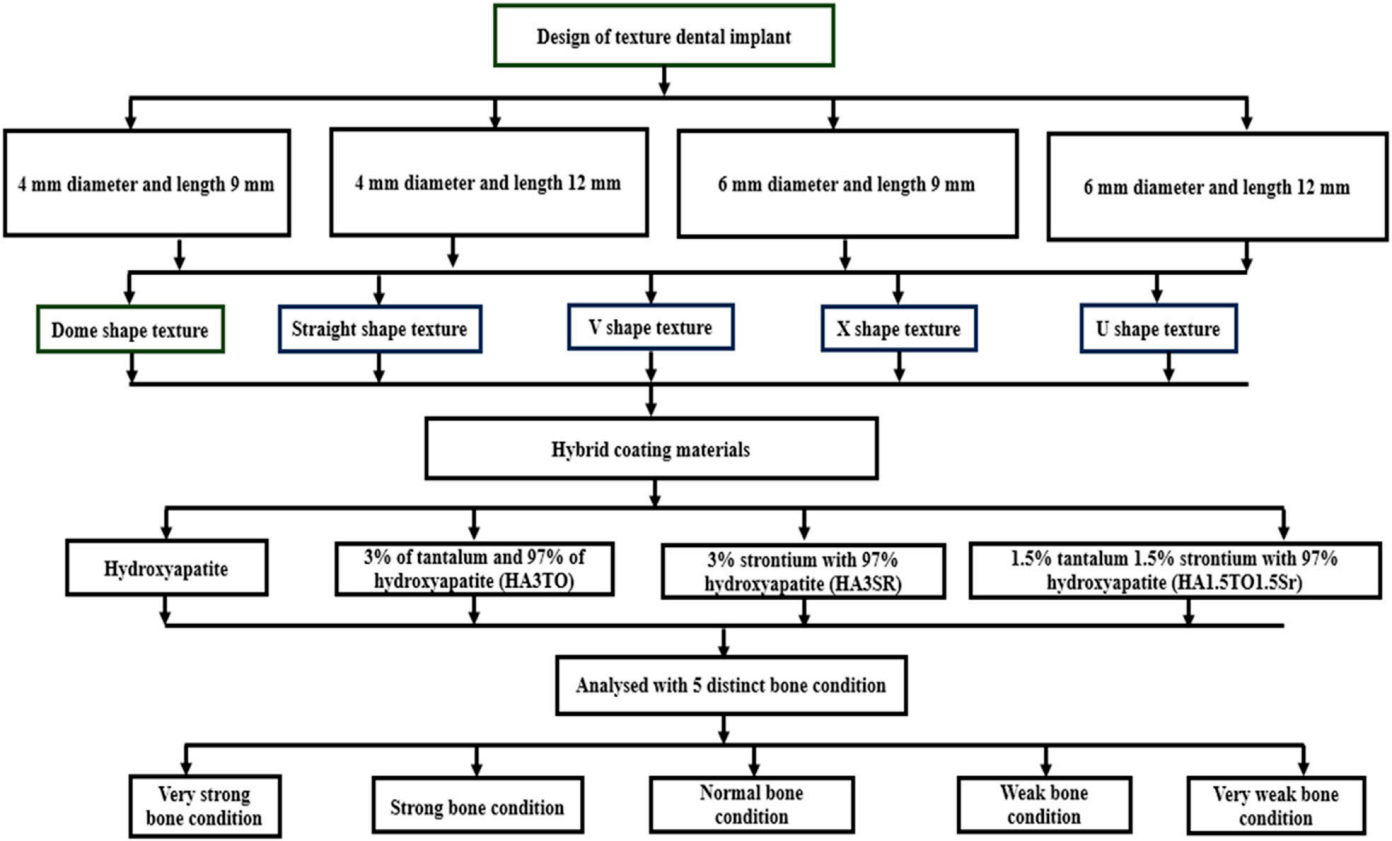
Flowchart for texture on dental implants with different bone conditions.

The coating materials are modeled with a 50 μm thickness using the sol-gel process (Dommeti et al., 2023b). Other solid models include abutments and crowns. SOLIDWORKS was used to design solid parts to be imported into the assembly module and constrained with respective surface-to-surface contact for all six solid parts. The SOLIDWORKS assembly models are imported into the Ansys space available in the geometry step in the static structural module. The flowchart of the entire modeling process is shown in Figure 2.

### 2.2 Mechanical properties

Prior to analysis, the mechanical properties of each dental implant component are considered as described in Table 1.

**TABLE 1:** Mechanical properties of different components.

S. No.	Component	Material	Young’s modulus (MPa)	Poisson’s ratio	
1	Crown	Cement	14,000	0.35	Kayabaşı et al. (2006)
2	Cap	Co-Cr	220,000	0.3	
3	Abutment	Surgical grade steel	187,500	0.33	
4	Implant	Titanium (Ti-6Al-4V)	110,000	0.32	
5	Gum	Gingiva	19.6	0.3	
6	Coating	HA	43,000	0.3	Dommeti et al. (2023b)
7	Coating	HA3TO	61,960	0.3	
8	Coating	HA3Sr	77,710	0.3	
9	Coating	HA1.5TO1.5Sr	57,210	0.3	

### 2.3 Analysis: loading and boundary conditions

**FIGURE 3:**
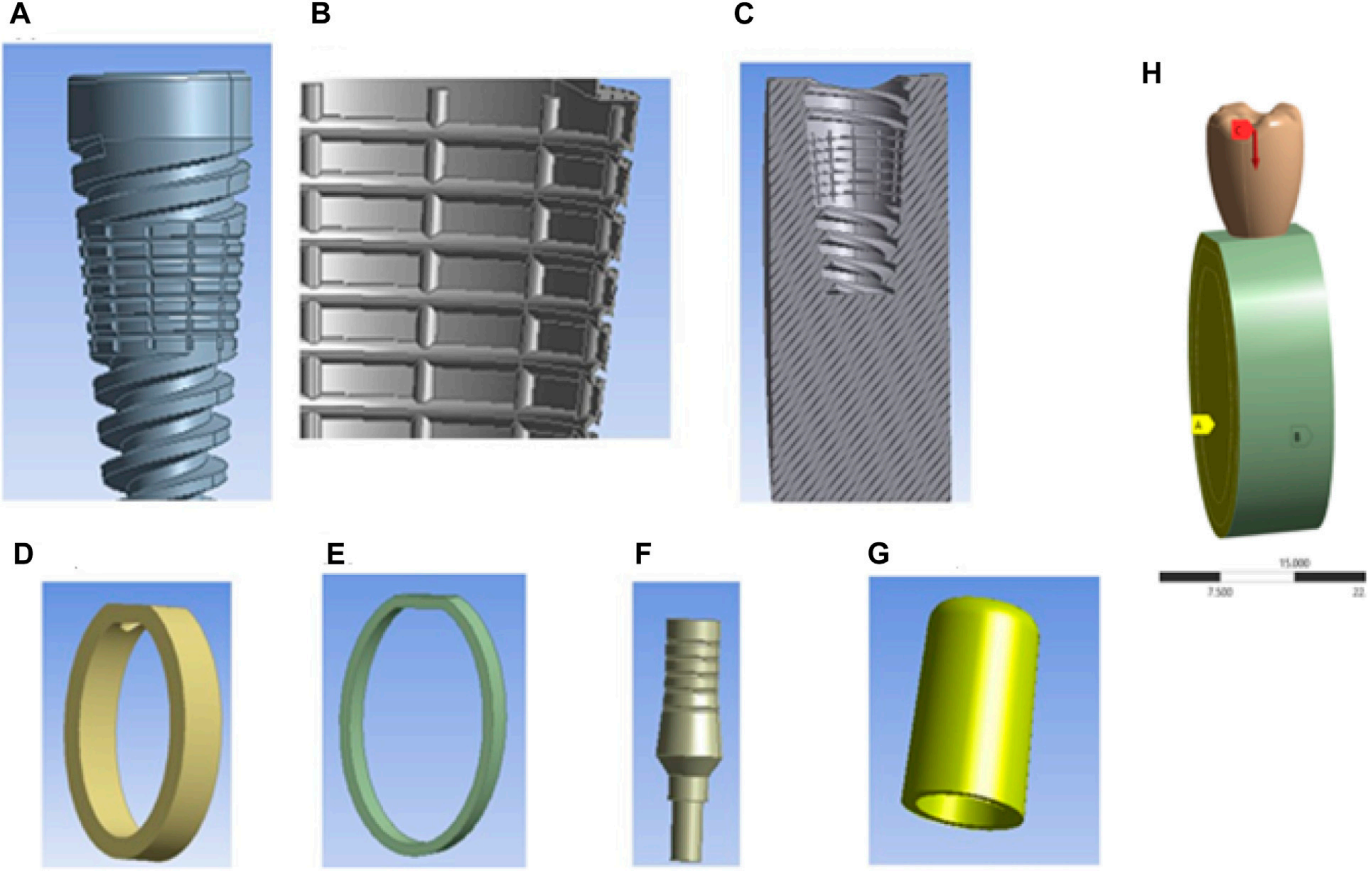
Components of textured dental implants and loading conditions: **(A)** Textured dental implant, **(B)** Coating material, **(C)** Cross-sectional view of cancellous bone, **(D)** Cortical bone, **(E)** Gingiva, **(F)** Abutment, **(G)** Cap, and **(H)** Assembly loading conditions.

The average biting force of 250 N is considered in the analysis (Roy et al., 2017). This load direction is vertical downward, and it is a point load. An anchor has been considered on both sides of the cortical bone, cancellous bone, and gingiva, and the analysis type is considered static in Figure 3. A mesh sensitivity analysis was carried out to validate the precision of the obtained outcomes. The mesh convergence study was executed using adaptive elements with sizes ranging from 0.1 mm to 1 mm to guarantee the precision of the numerical results. Finally, the mesh sizes were kept to a 0.2 mm element size for the overall assembled model structure after converging. The Solid 187 element was used in the study.

## 3 Results

This scholarly investigation systematically explores the biomechanical responses of dental implants, with a specific emphasis on the impact of surface texturing and varying bone quality. The study selects implants with diameters ranging from 4 mm to 6 mm and lengths of 9 mm and 12 mm to assess the influence of five distinct surface textures. This analysis is further enriched by evaluating the implants’ performance across five conditions of cortical bone quality, classified as very weak, weak, normal, strong, and very strong, with respective modulus values of 8,220 MPa, 10,960 MPa, 13,700 MPa, 16,440 MPa, and 19,180 MPa (Dommeti et al., 2024). A parallel assessment is conducted for cancellous bone, with conditions similarly categorized and assigned modulus values of 330 MPa, 440 MPa, 550 MPa, 660 MPa, and 770 MPa, respectively. The material properties pertinent to this investigation are detailed in Table 1.

The core objective of this study is to determine the stress distribution and interfacial strain between the textured implants and the cancellous bone tissue under physiological loading conditions. To this end, a loading force of 250 N, representative of typical masticatory forces, is applied to the implant. This approach facilitates a comprehensive understanding of how various surface textures interact with different bone qualities, thereby influencing the mechanical stability and osseointegration potential of the implants.

By integrating these parameters, the research aims to provide insights into the optimal design and surface engineering of dental implants, enhancing their clinical success rates in patients with diverse bone conditions.

**FIGURE 4:**
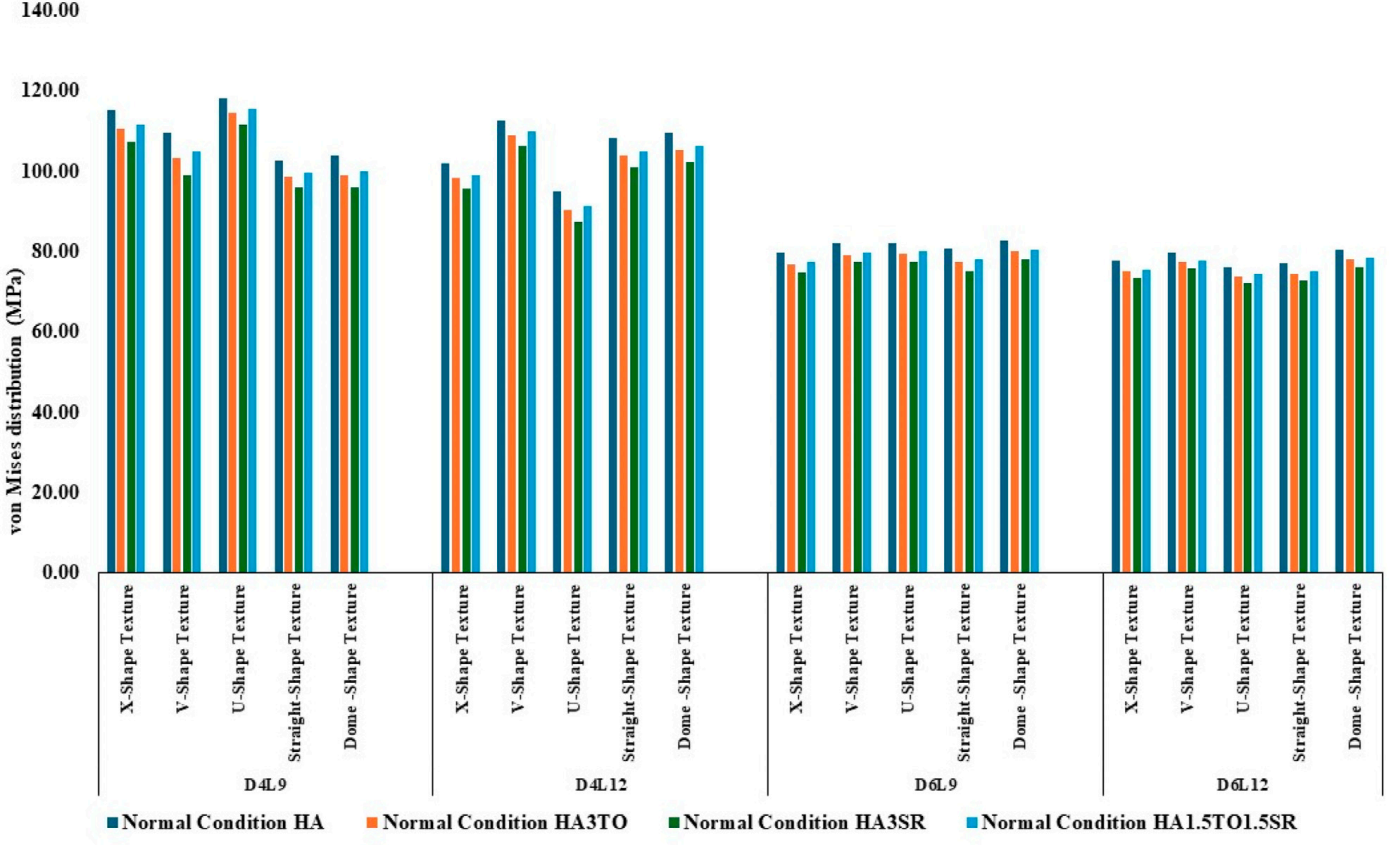
Comparison of stress values with coating interface stress and implants for the normal bone condition.

The von Mises stress distribution has been measured and is shown in Figure 4 for textured D4L9 implants. In normal bone conditions, the highest stress has been reported in the U-shape texture with the HA coating as 118.38 MPa. In the hybrid coating with 3% strontium, the stress was reduced by 5.83%, showing a gradual impact of introducing hybrid coating along with HA. In very strong bone conditions, the highest stress was reported in the U-shape texture with 117 MPa. However, it was reduced by 5.6% with a hybrid coating of 3% strontium. Similarly, the lowest stress was observed in straight texture with the same coating material. In other bone conditions, the highest stress was seen in the U-shape texture with HA coating material for strong, weak, and very weak bone conditions. Likewise, the lowest stress was found in the HA3SR coating combination when compared with HA; by adding strontium and tantalum, the stress was reduced by 21.3% for an implant with the straight texture.

**FIGURE 5:**
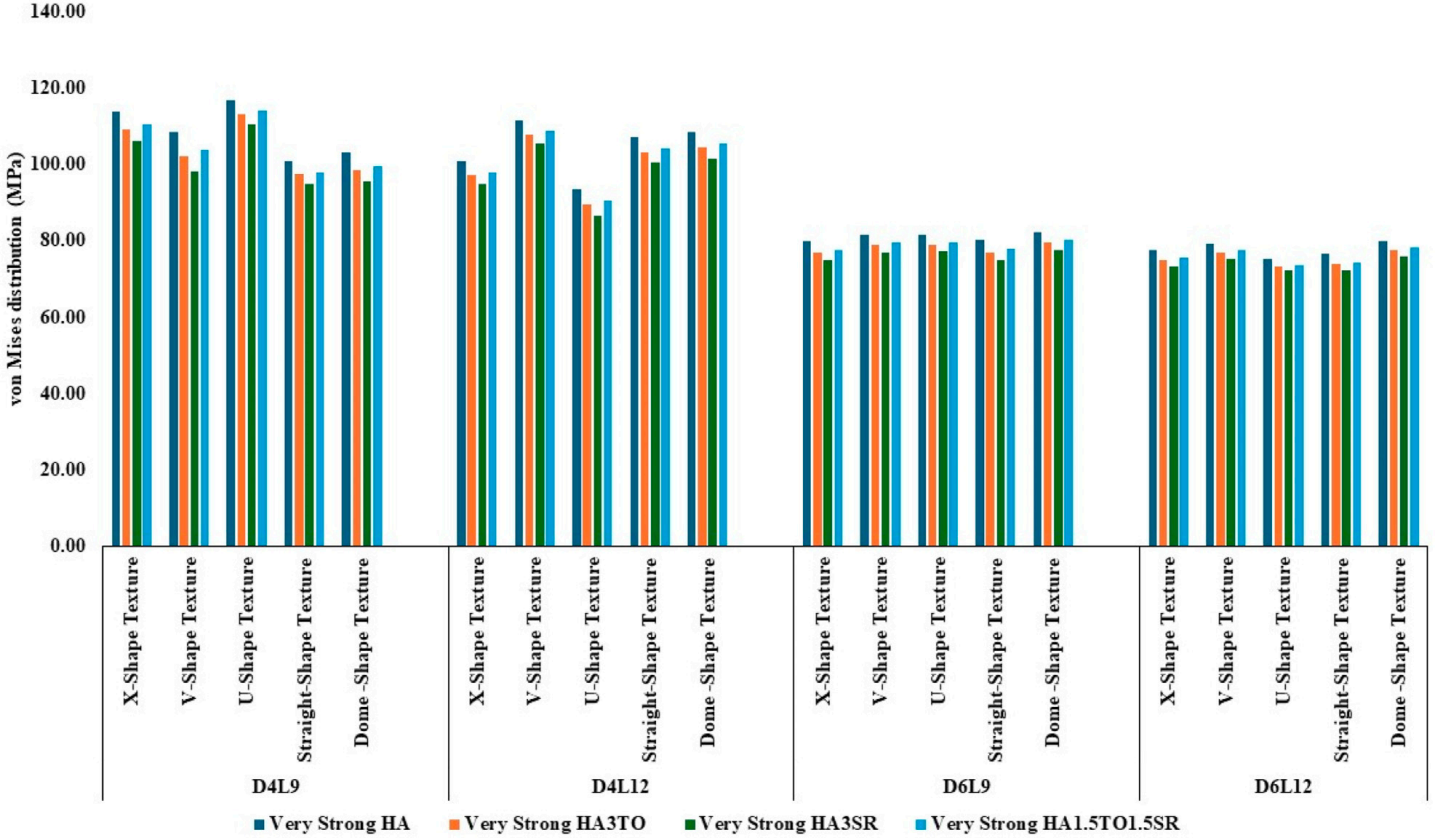
Comparison of stress values with coating interface stress and implants for the very strong bone condition.

In the case of D4L12 implants, as shown in Figure 5, at normal bone conditions, the highest stress was 112.50 MPa with the V-shape texture and the HA coating due to the sharp edges. The lowest stress was seen in the HA3SR coating and the U-shape texture, at 87.51 MPa, due to a larger contact area between the implant and the bone and the implant and the coating. In the same way as in other bone conditions, the U-shape texture showed lower stress with the 3% strontium coating. Compared with the HA coating, the stress in the implant was reduced by 28%.

**FIGURE 6:**
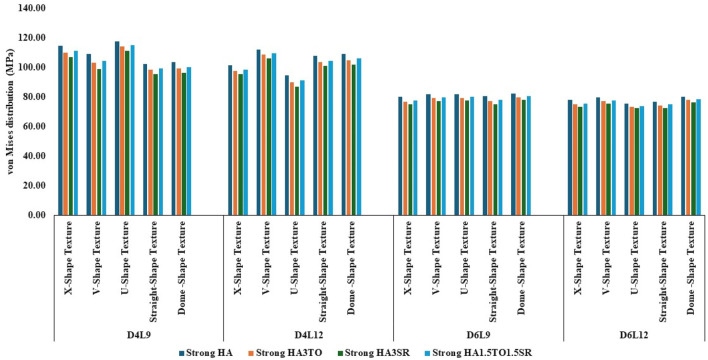
Comparison of stress values with coating interface stress and implants for the strong bone condition.

In the case of D6L9 implants, the maximum stress in the normal condition, 82.61 MPa, was seen with the U-shape texture and the HA coating material. The results are reduced by 7.8% for the strontium and tantalum combination seen in HA3SR. For very strong bone conditions, the U- and V-shape textures with the HA coating and the straight and X-shape textures with the HA3SR coating show the minimum stress. In the case of the strong bone condition, as shown in Figure 6 maximum stress was observed with the dome shape and HA coating, and the minimum stress was observed for the straight and V-shape textures with the H3SR coating. For weak and very weak bone conditions, the maximum stress in the dome shape in HA coating material, and the lowest stress is the X-shape texture with the strontium coating, with a 9.7% reduction.

**FIGURE 7:**
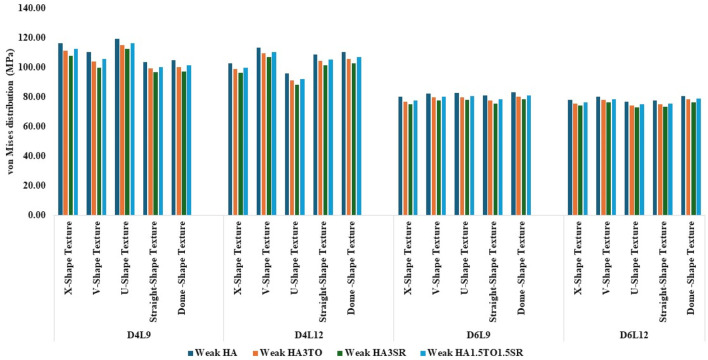
Comparison of stress values with coating interface stress and implants for the weak bone condition.

In weak bone conditions, as shown in Figure 7 the study’s findings indicate that the U-shaped coating with hydroxyapatite (HA) exhibited the highest implant stress of 119 MPa, while the dome and straight configurations showed lower stresses of 96.96 MPa and 96.44 MPa, respectively. Conversely, in the D4L12 implants, the maximum stress was observed in the V-shaped texture, attributed to its sharp edges, with a value of 113 MPa. The U-shaped texture, on the other hand, demonstrated the lowest stress of 87.98 MPa when coated with HA3SR.

**FIGURE 8:**
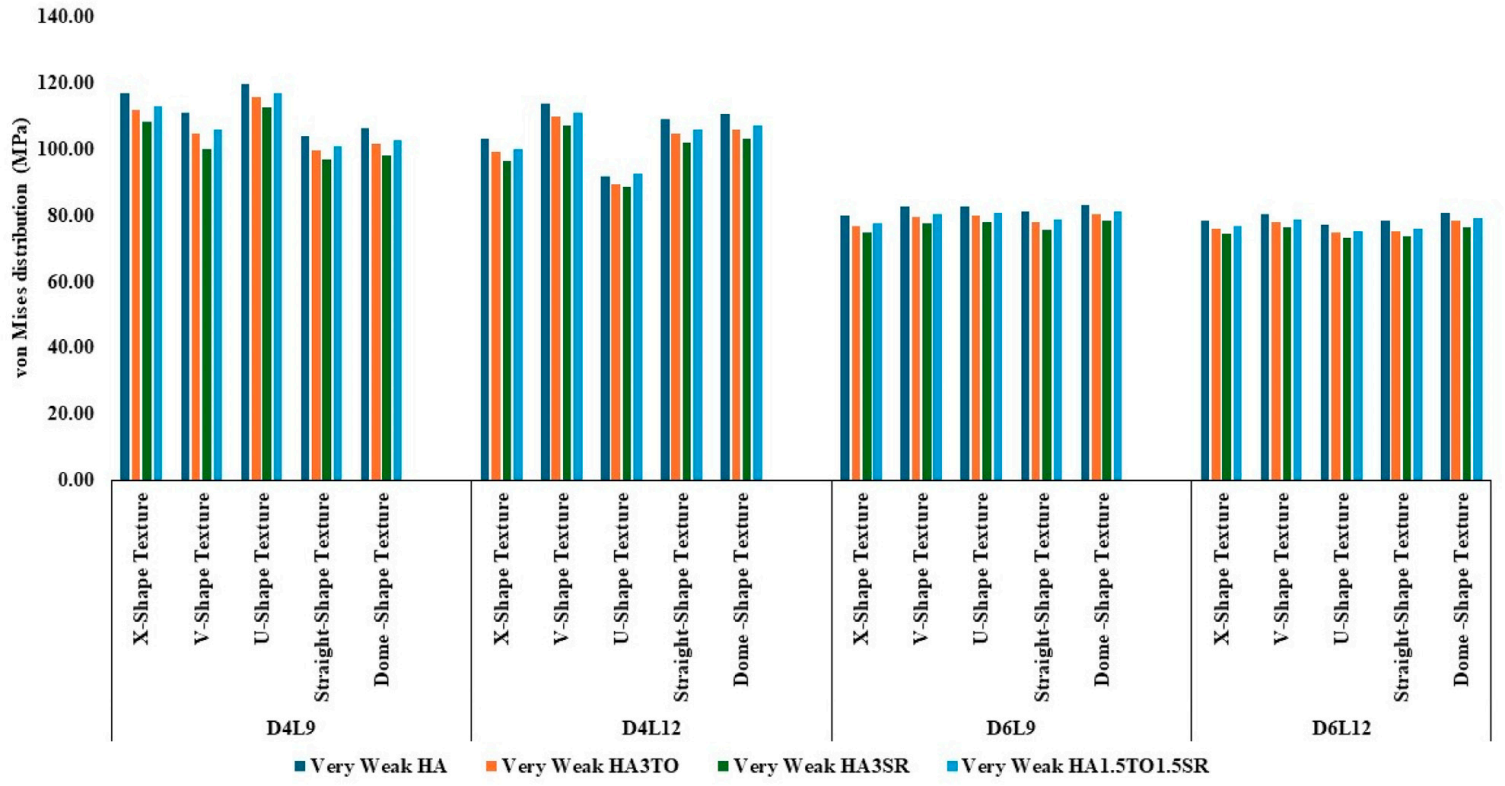
Comparison of stress values with coating interface stress and implants for the very weak bone condition.

In very weak bone conditions, as depicted in Figure 8, The hydroxyapatite (HA) coating exhibited higher stress levels than the hybrid HA3SR and other coatings, with a 21% increase observed particularly in the straight shape texture. Conversely, the U-shaped texture coated with HA3SR and HA1.5TO1.5SR showed lower stress levels than the V-shaped texture, with a reduction of 24.8% noted in the hybrid coating. Moreover, in the cases of the larger diameters examined in the current study, stress distribution appeared to be more uniform.

**FIGURE 9:**
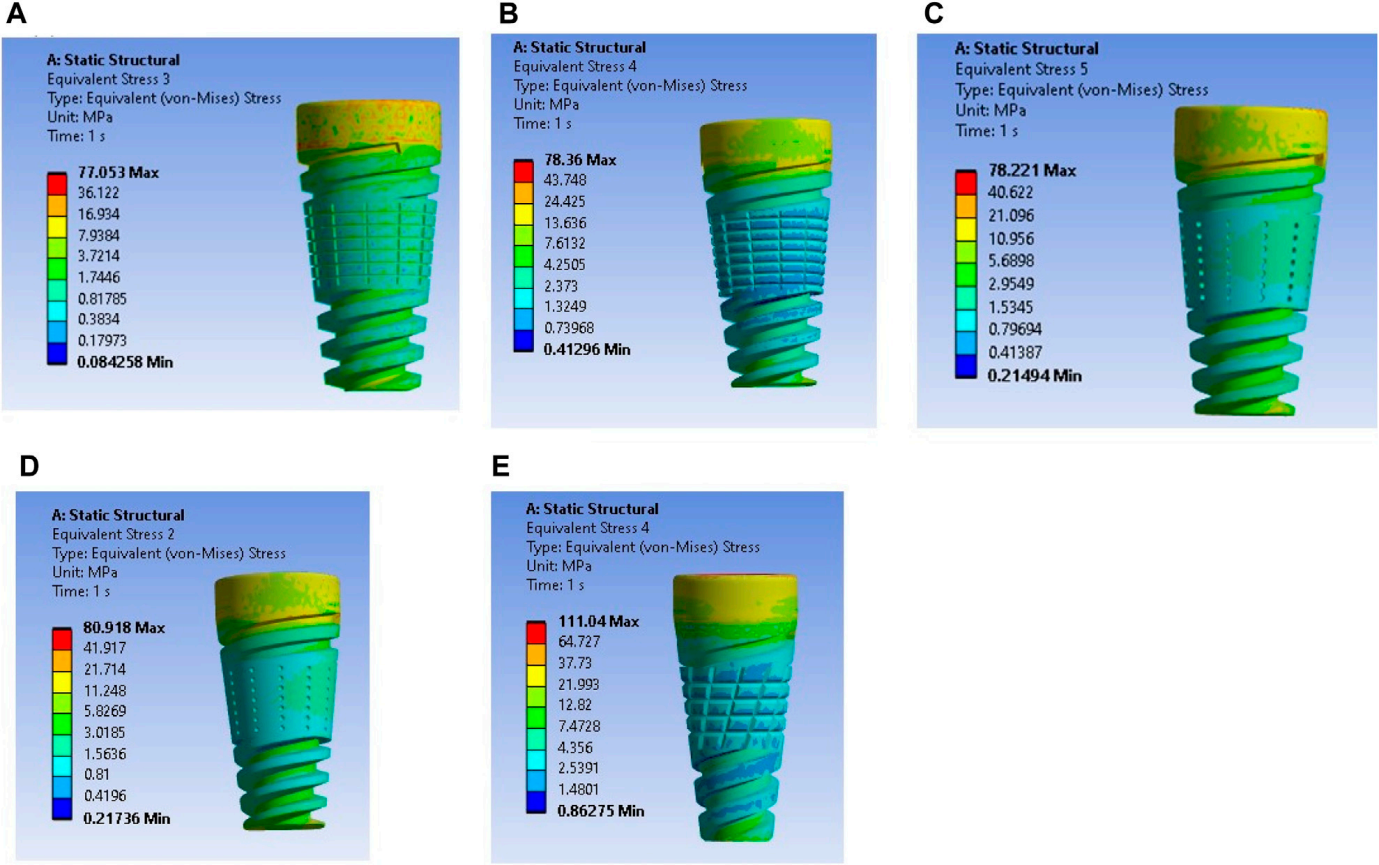
Stress induced in a textured implant with a weak bone condition: **(A)** U-shape texture with D6L12, **(B)** V-shape texture, **(C)** Straight shape texture, **(D)** Dome shape texture, and **(E)** X-shape texture.

The maximum stress values were observed in the very weak bone condition with a dome-shaped texture, registering at 80.92 MPa. The results underscore the significance of strong bone quality in ensuring enhanced stability for the implant and reduced stress at the bone–implant interface. Conversely, weaker bone conditions are associated with less stable implant placements, heightened stress concentrations, and an increased risk of implant failure, as depicted in Figure 9.

### 3.1 Interfacial strain induced in cancellous bone

A thorough examination of the strain occurring at the bone–implant interface is essential when comprehensively investigating the effects of bioactive implants. It is critical to emphasize that interfacial strain plays a pivotal role in this analysis.

**FIGURE 10:**
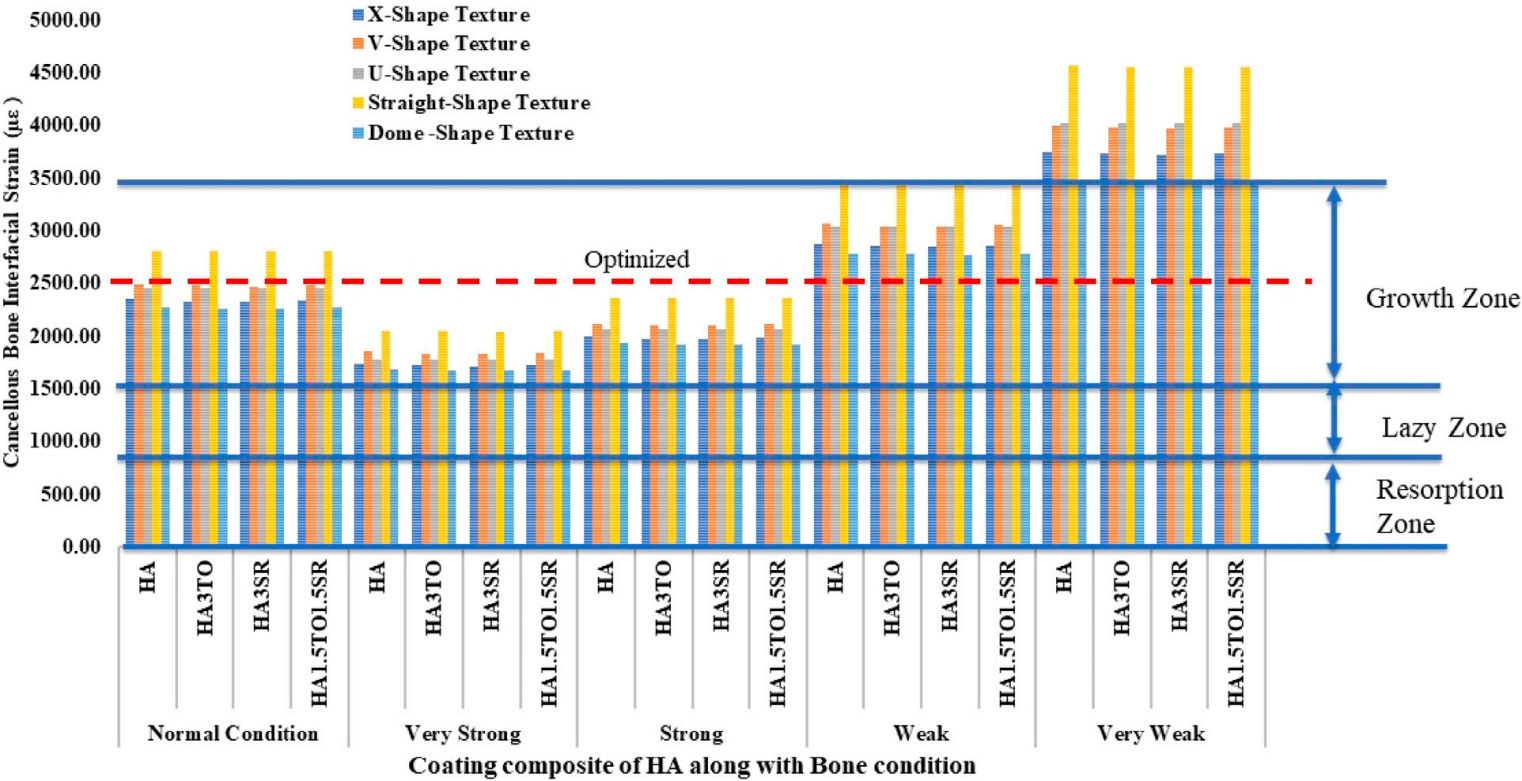
Interfacial strain induced in a D4L9 implant for all bone conditions.

Figure 10 illustrates the mechanostat hypothesis, providing interfacial microstrain values (1,500–3,000 µε) for different textures. In very weak bone conditions, the dome shape texture shows a microstrain of 3,473 µε in the growth zone, which is higher than other textures. All microstrain values fall within the growth zone for normal, very strong, strong, and weak bone conditions.

**FIGURE 11:**
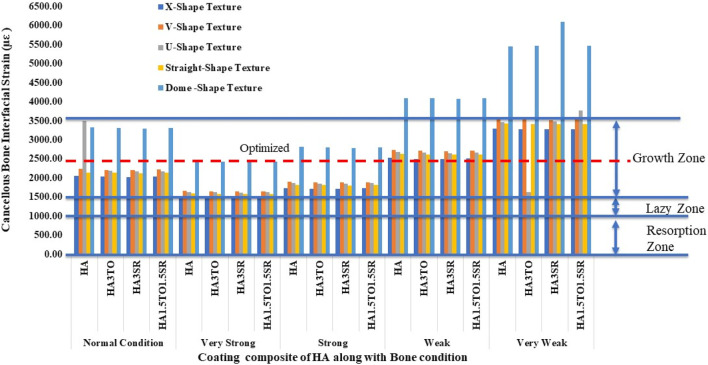
Interfacial strain induced in D4L12 implant for all bone conditions.

For the 6D9L implants, the X-shape texture is shown above the growth zone, while V-shape, U-shape, straight, and dome textures are within the growth zone. In weak and very weak bone conditions, these textures exhibit better results, as depicted in Figure 11.

**FIGURE 12:**
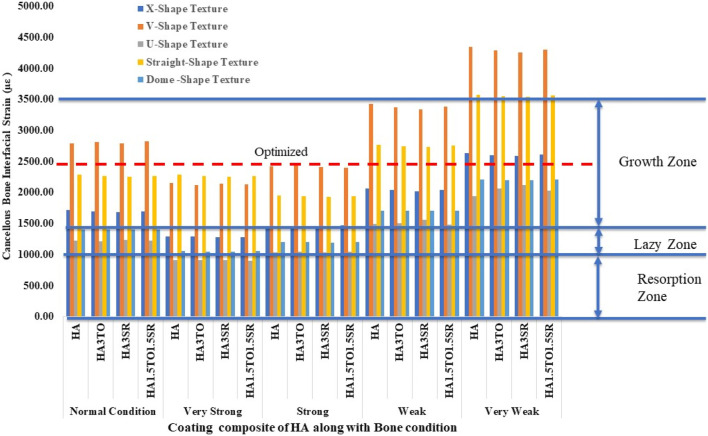
Interfacial strain induced in D6L9 implant for all bone conditions.

For the 6D9L implants, an increase in diameter results in the U-shape, straight, and dome shapes being in the growth zone. Particularly in very weak bone conditions, the U-shape texture exhibits a maximum microstrain of 2035 µε with the HA1.5TO1.5SR coating combination, as illustrated in Figure 12.

**FIGURE 13:**
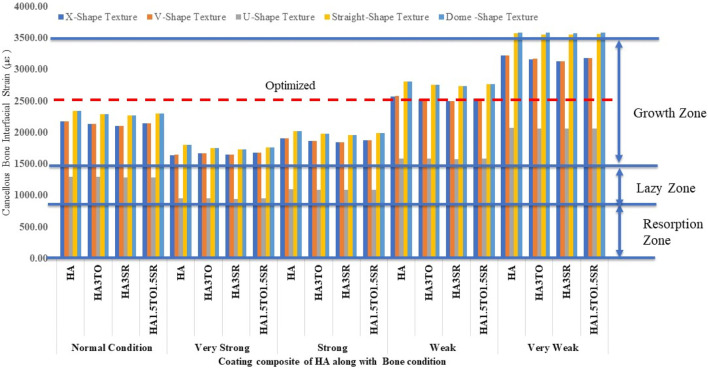
Interfacial strain induced in a D6L12 implant for all bone conditions.

For the 6D12L implants, U-shape textures are located in the lazy zone in normal, very strong, and strong bone conditions. However, in weak bone conditions, the microstrain is 1,583 µε with an HA coating, and in very weak bone conditions, it reaches a maximum of 2075 µε with an HA coating material, as shown in Figure 13.

## 4 Discussion

The distribution of stress and strain in the bone surrounding osseointegrated dental prostheses is influenced by various biomechanical factors. These factors include the geometry of the implant screw, the material properties of the prosthesis, the type of loading applied, the quality of the surrounding bone, and the condition of the bone-implant interface.

In this study, a model with five different surface textures and four different coatings was simulated, aiming to fill gaps between the bone and the implant. Solid 4D12L implants without coating were compared with the current work, showing a reduction of about 19% (Kayabaşı et al., 2006).

For HA3SR-coated implants in very strong bone conditions, the stress value was 86.60 MPa, which is an 8% reduction compared to previous work involving textured and coated implants (Dommeti et al., 2024).

### 4.1 Effect of coating on dental implant

The stability of dental implants, encompassing both primary and secondary stability, significantly influences the success of osseointegration (Inchingolo et al., 2021). Hydroxyapatite coatings have been found effective in promoting osseointegration, with various studies highlighting their benefits in enhancing implant stability and bone formation, often through methods like sol-gel (Kim et al., 2021; Neto et al., 2023). For optimal remodeling, it is ideal for implant strain to fall within the 1,500–3,000 microstrain range. This range facilitates appropriate bone remodeling without causing excessive strain, thus promoting favorable long-term outcomes. Therefore, selecting implants that minimize stress while keeping strain within this range is crucial for stability and remodeling processes (Aversa et al., 2009). Previous reported work indicates that implants exhibiting interface strain within the growth zone of 1,500–3,000 microstrain show the best bone remodeling and osseointegration.

**FIGURE 14:**
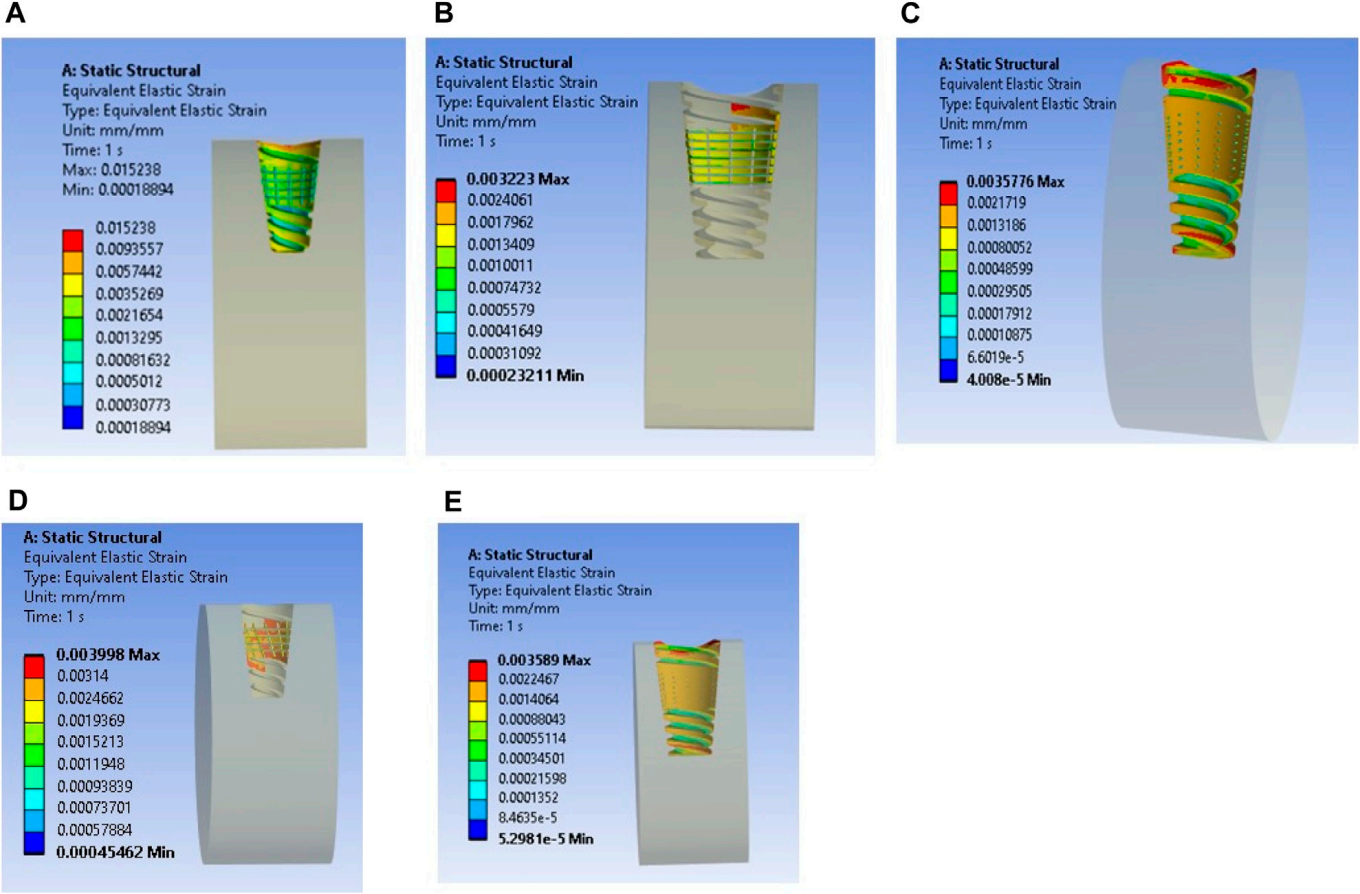
Interfacial strain for textured dental implant with coating with weak bone condition: **(A)** U-shape texture, **(B)** V-shape texture, **(C)** Straight texture, **(D)** X-shape texture, and **(E)** Dome shape texture.

The results closely align with previous research, indicating an optimal microstrain of 2,500 for various lengths and diameters, as depicted in Figure 8 (D4L9), with all textures considered in weak and very weak conditions. For D4L12 implants, as shown in Figure 10, the U-shape texture is in the growth zone for normal, strong, weak, and very weak bone conditions. However, for weak and very weak bone conditions, all textures and coatings exhibit microstrain values above optimal, except for the dome shape texture, which is shown above the growth zone and can be disregarded. In the case of D6L9 implants, as illustrated in Figure 10, the straight texture and V-shape texture with HA coating fall within the optimized zone, while other textures support the growth zone. However, the straight texture with all coatings is shown above the growth zone and may not be considered. Figures 11, 14 show that D6L12 implants exhibit better microstrain results in weak and very weak bone conditions for all bone conditions. When compared with solid implants, implants with texturing, surface treatments with 3D-TIPS in 3D printed implants, and nano-scale technological advancements on titanium implant surfaces to enhance the osseointegration process, the results indicate advancements in implant technology (Roy et al., 2017; Lee et al., 2021).

## 5 Conclusion

The present study investigates the influence of different implant surface textures on various bone qualities, focusing on dental implants with diameters of 4 mm and 6 mm. For weak and very weak bone conditions, surface texture modifications on dental implants, compared to solid implants, show optimal microstrain results with all textures, particularly with HA and HA1.5TO1.5Sr coatings for D4L9 implants.

For D4L12 implants, weak and very weak bone conditions exhibit better results with all textures except for the X-shape texture. In the case of D6L9 implants, the U-shape and X-shape textures demonstrate superior results. However, for D6L12 implants, textures with straight and dome shapes are not suitable for weak and very weak bone conditions.

While stress levels may be elevated for certain implants in bones with specific conditions, all stress values remain within acceptable limits for the mandible.

## Data Availability

The raw data supporting the conclusions of this article will be made available by the authors, without undue reservation.
